# Experimental Investigation and CFD Modeling of Slush Cryogen Flow Measurement Using Circular Shape Capacitors

**DOI:** 10.3390/s20072117

**Published:** 2020-04-09

**Authors:** Bogdan Florian Monea, Eusebiu Ilarian Ionete, Stefan Ionut Spiridon

**Affiliations:** National R&D Institute for Cryogenics and Isotopic Technologies ICSI Rm. Valcea, 240050 Ramnicu Valcea, Romania; bogdan.monea@icsi.ro (B.F.M.); ionut.spiridon@icsi.ro (S.I.S.)

**Keywords:** cryogenic fluid, slush flowmeter, circular capacitors, numerical simulation

## Abstract

The measurement of two-phase cryogenic fluid mixtures flow, also known as slush cryogen flow, with its most attractive form (liquid and solid) is of great interest for various applications, due to its thermodynamic advantages. This paper presents a newly developed device, under the form of a circular capacitor prototype, together with an experimental stand for slush formation. Slush nitrogen was used as testing fluid during the experimental work. Then, the experimental data for slush cryogen flow measurement using the proposed circular shape capacitor were compared with theoretical results obtained by simulation. A three-dimensional flow field model was built and solved for the innovative design slush flowmeter using a computational fluid dynamic (CFD) model. Nitrogen slush density of 874 kg/m^3^, representing approximately 30% solid fraction, was reported for both the modeling and experimental testing, although the numerical investigation is not limited to these values. By comparing experimental vs. simulation results, a deeper view on the designed configuration can be achieved, thus improving the progress in producing high-performance next generation devices for two-phase flow measurement in terms of physical dimensions, length and space between armatures. Even so, the mathematical model has limitations when mixtures with higher percentages of solid phase and particle sizes are encountered.

## 1. Introduction

Two-phase combinations (liquid–gas or liquid–solid) of the same matter [1] travelling together, in the same place, at the same time, represent a two-phase flow. In the particularly field of cryogenics, liquid–solid mixture, composed of fine solid particles dispersed in the liquid phase, is the most usual, sought after and interesting combination. Occasionally liquid–solid flow can be seen from slight freezing of the liquid, but the primary two-phase flow seen in cryogenics domain is liquid–gas [1]. Unusually, during gas liquefaction processes, when the production of the liquid–solid mixture is not within the objective of that cryogenic installation, it can be formed for a short time and in an uncontrollable, unstable manner. Since the solid–liquid mixtures occur after a gas liquefaction process, practically after reaching the normal boiling point of that substance, when considering the cryogenic domain, the most common biphasic mixture is the liquid–gas one. 

On the other hand, liquid–solid mixtures—generally known as slush cryogen—present a number of advantages such as increased capacity to absorb heat in comparison with the liquid form, and higher mass transfer rate due to higher density than liquid at normal boiling point [2]. The most attractive forms are slush nitrogen and slush hydrogen, which have attained large popularity lately, although other forms do exist, such as slush oxygen. This interest is tributary to several technological applications using cryogenic flow systems that include the possible usage of slush as fuel for rockets in the US, the European Ariane 5 program, or for the reusable space shuttle in Japan [2]. By producing and using slush hydrogen, since mixture density is higher than liquid density and combined with increased heat capacity, the resulting physical dimensions for the rocket fuel tank are smaller for the same hold-up. 

The main advantages of using slush hydrogen for space program applications [3], as compared to the usage of its liquid state, are related to its high density and reduced fuel loss through evaporation. To take full benefits of these advantages, the slush solid content must be at the maximum that can be used. To achieve maximum concentrations of solids in slush hydrogen, the mass solid fraction will need to be continuously or periodically increased in ground or orbital storage as well as in flight vessels [4].

Despite all these advantages some problems still exist and are related to the slush hydrodynamic characteristics, low density and mixture holding time due to low temperature. Lately, new discoveries and advances in the field of superconductive materials, with alloys presenting superconductivity properties at temperatures way higher than that of boiling point, have opened the door for the extensive usage of slush nitrogen as cooling fluid. Thus, several new applications have been identified [5,6], e.g., as refrigerant for long distance superconducting cables, cables capable to sustain very large currents over very long distances.

In this paper we report several tests on slush cryogen flow measurement using a circular shape capacitor in an innovative configuration. The new proposed configuration was based on the limitations that we have encountered with the previous configuration that we have studied [7]. The main advantage is related to the clogging situation, when the flow was blocked due to the presence of big ice chunks, phenomena that was not encountered with this design. The ice chunks with uneven shapes are stopped, by the cone-shaped configuration itself, before entering between the capacitor’s armatures. Additionally, to better understand the flow phenomena within the experimental device, a numerical model was built and solved using the computational fluid dynamics (CFD) software package Fluent, considering several working conditions similar to those in experiments. A CFD analysis is an important tool in today’s engineering and can be used in various fields from the simulation of the development of a chemical sample collection device inspired by a crayfish [8] to size distribution and concentration of airborne Martian dust [9]. Slush nitrogen was selected as investigated fluid due to its ease production way and safety matters.

## 2. Apparatus and Methods

### 2.1. Cryogenic Fluid Flow Measurement

There are several methods (i.e., Coriolis, turbine, Venturi flowmeters, flowmeters based on acoustic effect and based on Doppler effect, flowmeters based on the introduction of a temperature pulse, V-type flowmeters, etc.) widely used for measuring cryogenic fluid flow rates. 

Given the particularities of cryogenic fluids and especially those related to very low working temperature, not all devices and methods for measuring fluid flows, imagined so far and available on the market or known to the scientific world, can be used for measuring cryogenic fluid flows. Furthermore, due to the fact that solid–liquid mixture presents other characteristics, in addition to those related to very low temperatures, the methods of measuring the flow rates of cryogenic biphasic solid–liquid mixtures are limited to only a few. Ohira, Nakamichi and Kihara [10] developed a wave-type flowmeter using a microwave method for slush hydrogen, and two other research groups presented configurations of the used flowmeters [7,11].

### 2.2. Principle of the Capacitance-Type Flowmeter

**Figure 1:**
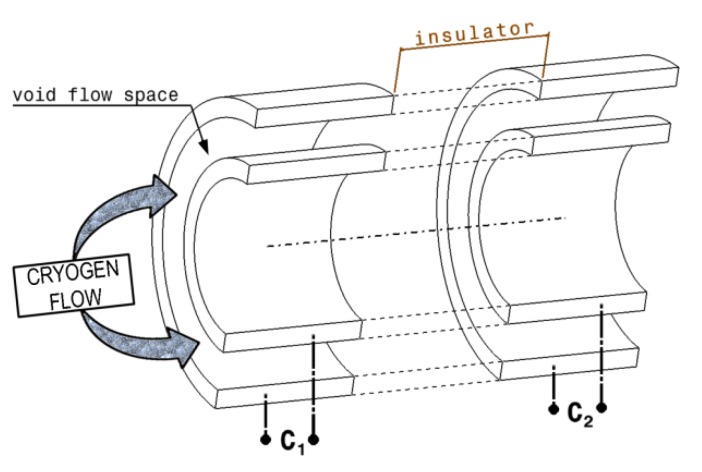
Schematic view of a capacity type flow meter.

The basic configuration of a capacitance-type flowmeter (Figure 1) consists of two identical capacitors mounted on the same flow of cryogen, having a very well determined distance between them [7,12].

Due to the changes occurring within the two-phase flow, generated by the uneven distribution of solid particles within the mixture flow, the density of the cryogen can fluctuate rapidly, fluctuations that can be detected by the two capacitive devices. The mass flow rate, since the capacitance-type flowmeter is a mass flow meter, is:

*Q_m_* = *ρ_sl_* · *v* · *S*, (1)

where *Q_m_* is the mass flow rate, *ρ_sl_* is the density of slush cryogen, *v* is the flow velocity and *S* is the cross-section area of the flow, determined by mechanical design.

The fluid velocity is measured inside of the capacitance type flowmeter, using the distance between the capacitors *C1* and *C2* (Figure 1) and the delay time (*τ*) necessary for the fluid to travel this distance. The delay time (*τ*) can be determined using the cross-correlation function (*R*(*τ*)) of two signals, *X*(*i*) and *Y*(*i*) [13]. *X*(*i*) and *Y*(*i*) are signal values representing capacity variations measured by first and second capacitor, respectively, expressed as a function of time. The function *R*(*τ*) reaches the maximum when *τ* becomes equal to the real transit time of a density pulse (i.e., the time required by a density peak detected by the first capacitive sensor to be also detected by the second capacitive sensor) [14]. The cross-correlation function *R*(*τ*) is defined as:

(2) $$R\left(\tau \right)=\int_{0}^{t}X(t)Y(t-\tau )\mathrm{dt},$$

### 2.3. Principle of the Capacitance-Type Density Measurement

Since the dielectric constant of a cryogen, which can be considered ideal, homogeneous and isotropic obeys the Clausius–Mossotti law [12], the density measurement of two-phase mixture can be determined with capacitive methods based on the equation [14]:

(3) $$\frac{\varepsilon -1}{\varepsilon +2}\rho^{-1}=P,$$

where *ε* is the relative dielectric permittivity of the mixture and *P* is the polarizability.

It has been experimentally found that polarizability present dependencies on density *ρ*, temperature *T* and pressure *P*. The equation modeling the polarizability includes high order correction terms, with constants coefficients that are unique to the cryogen of interest [15]. Polarizability coefficient experimentally measured increases with about 0.1% when hydrogen changes from liquid to 0.5 solid fraction at triple point [16].

For simplicity, in our experiments we considered the polarizability factor as being constant, so the dielectric permittivity is the unique factor influencing the density. Since the density and the specific dielectric permittivity of the liquid and solid phases have already been determined (see Table 1), the slush density (*ρ_sl_*) can be determined from Equation (3), where the specific dielectric constant of slush fluid can be measured from the capacity Equation (4) (see below).

Further, after determining the slush density we can determine the volume fraction of solid phase (*a_s_*) with the relation:

(4) $$a_{s}=\frac{\rho_{sl}-\rho_{l}}{\rho_{s}-\rho_{l}}\times 100,$$

### 2.4. Experimental Setup

**Figure 2:**
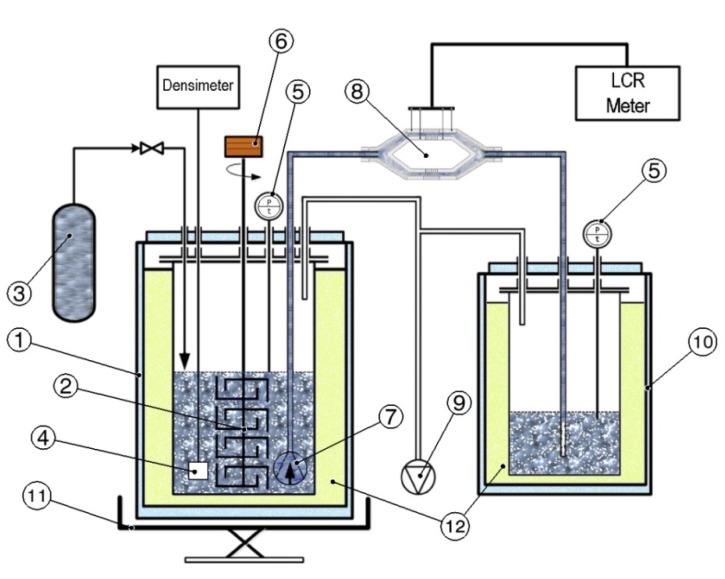
Schematic of the experimental set-up of slush flowmeter test rig: 1—Dewar slush forming, 2—stirrer, 3—liquid nitrogen tank, 4—densimeter, 5—vacuum and temperature measurement, 6—electric stirrers motor, 7—cryogenic slush transfer pump, 8—flowmeter, 9—vacuum pump unit, 10—receiver Dewar vessel, 11—weighing scale, 12—Dewar’s thermal insulation.

Slush nitrogen obtained through a self-pressurized freeze-thaw method was used in our experiments. This method it resumed as a continuous evacuation of the nitrogen vapors during the freeze cycle, creating in this way solid layers on the liquid/vapor interface, that subsequent submerge into the liquid during the thaw cycle process. The experimental setup is schematically illustrated in Figure 2 and is based on the one previously described in [13].

The vacuum pump unit ⑨ connected to the Dewar vessel, the slush main storage and production container, is used to evacuate the nitrogen vapors in order to reduce the inside pressure and to form solid layers. The process takes place by removing the latent heat of vaporization and by moving thermo-physical state of nitrogen bath along the saturated vapor pressure curve, from 77 K to 63.15 K [19,20]. Additionally, the installation contains a pressure gauge and temperature sensors ⑤ to determine the temporary reached temperature and proper pressure controlling systems with necessary valves. The slush nitrogen producing and receiving Dewar vessels are protected with a very good cryogenic thermal insulation. A stirrer (mixing impeller) ⑥ is installed on top of the producing Dewar vessel and is used to break the nitrogen ice, after the formation of solid nitrogen on the liquid free surface, to a desirable size range and to its uniform distribution inside. 

During the production process, when the desired nitrogen ice quantity is reached (measured with the densimeter ④, made under the same shape, sizes, materials and technology as the flowmeter capacitors), the stirrer is actuated to determine the shredding of ice clusters to the desired size, thus forming the slush nitrogen, small solid particles, homogenous in the whole liquid bath. Slush flow to the pipes and flowmeter is determined by the transfer pump ⑦ immersed in the slush bath.

**Figure 3:**
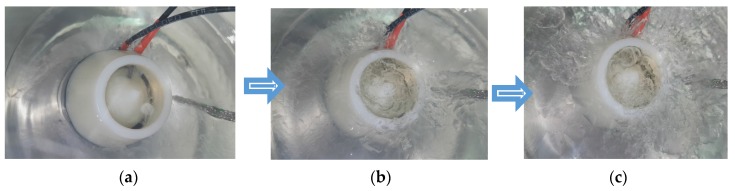
Picture of the reference capacitor inserted in slush nitrogen during different stages of the freezing cycle: (**a**) after 20 s; (**b**) after 50 s; (**c**) after 100 s.

Finally, the freeze and thaw selected cycle time was chosen between 120 s and 60 s in order to obtain small solid particles, homogenous in the whole liquid bath. Figure 3 presents images of different stages of slush formation.

### 2.5. Flowmeter Design

Several sets of experiments were performed using slush nitrogen with the new proposed design of capacitance type flowmeter for two-phase mixtures of cryogenic fluids, design that is protected by a patent [21]. The investigated design assures a good insulation between the armatures and good mechanical integrity at low temperature. Capacitor plates are of circular shape, differing from our previous presented design [7] and from other designs presented in literature [22]. 

**Figure 4:**
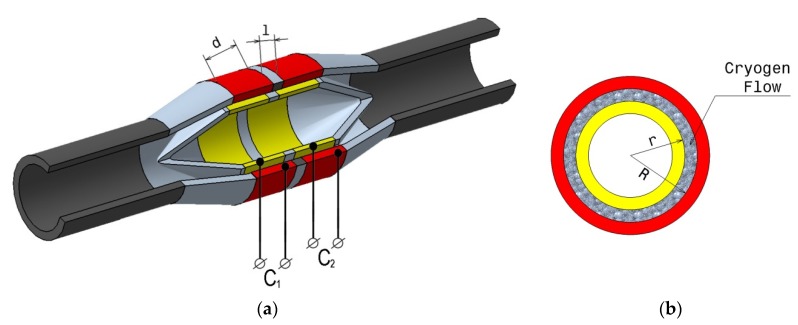
(**a**) Cross section schematic design of the capacitance type flowmeter; (**b**) cross section of one of the capacitor.

**Figure 5:**
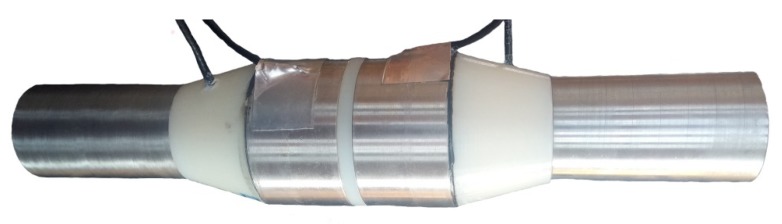
Image of the experimental device.

A cross section of the used device is presented in Figure 4 and the image of the experimental device in Figure 5. It consists in a succession of two cylindrical capacitors placed on the fluid flow stream, at a certain distance between them.

The operating principle of this flow meter is based on forcing the flow of the two-phase mixture, without requiring elements to calm the turbulence of the flow, between the armatures of circular capacitors, arranged successively, and electrically isolated from each other. The annular capacitors consist of two collinear cylinders of different diameters, arranged concentric with a well determined distance between them. Forcing the flow of the two-phase mixture of cryogenic fluid between the armatures of the capacitors is done by means of a cone type element with a hemispherical shape included in another cone (cone trunk) having at the top the inlet tube, being disposed within the flowmeter body and which makes so that the fluid is evenly distributed between the capacitor armatures. The restoration of the flow profile of the fluid, after it has passed through the flowmeter (the succession of the two capacitors), is done with a cone type element, similar to the one at the input, but disposed in reverse configuration. In addition, these elements also act as an electrical insulator between the inlet/outlet tube and the capacitor armatures.

A first step in manufacturing the flow meter consisted of the realization of the cone type elements. These elements were made from thermoplastic aliphatic polyester (PLA), using a 3D printer. The cylinders of the two capacitors and the inlet and outlet tubes were made of stainless steel. The electrical connections for measuring the capacity values were made using coaxial cables, type RG179, double shielded, having a total thickness of 2.54 mm and a small parasitic capacity (approximately 57 pF/m). After the electrical connections were made, all the constituent elements of the flow meter were assembled; particular attention was paid to the gluing process with a special cryogenic adhesive on the stainless-steel support (Figure 5).

The physical dimensions of the realized experimental device are as follow: total length, 245 mm; inlet/outlet tube diameter, 28 mm; capacitors length, *d* = 30 mm; outer cylinder inside radius, *R* = 22 mm; inner cylinder outside radius, *r* = 19 mm; distance between *C1* and *C2* is *l* = 5 mm. The capacitors face (the sensors faces), exposed to the fluid flow, and were polished to the mirror like finishes before the start of the assembly process. 

From the capacitance relation of a capacitor having circular armatures:

(5) $$C=\frac{2\pi \varepsilon_{o}\varepsilon_{r}d}{\ln\left(\frac{R}{r}\right)},$$

was determined *ε_r_*, and further, using relation (3) the density of the mixture was calculated.

## 3. Experimental Results

Before the slush nitrogen flow experiments a calibration of the capacitors was made by measuring their values in air at normal conditions (room temperature and pressure) and immersed in nitrogen at normal boiling point (NBP) and at triple point (TP) (Table 2). It can be noticed that differences between the capacitor’s calibration values are presented, mostly due to the installation method or their geometric shape (cross-sectional area).

**Figure 6:**
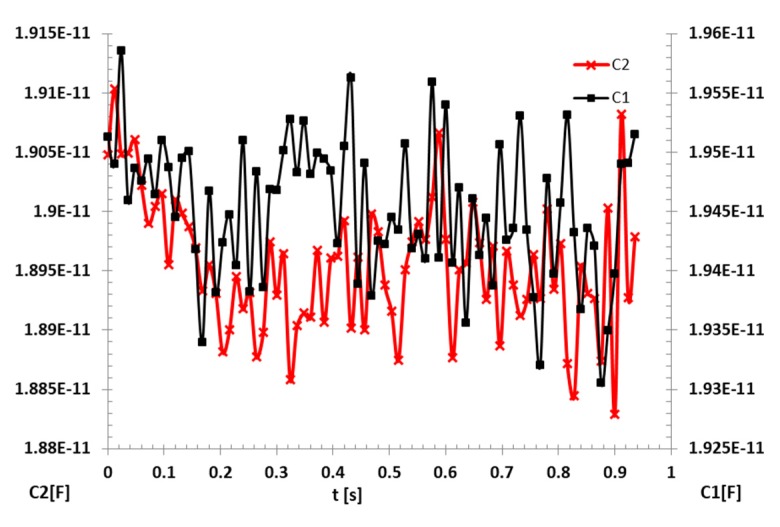
The evolution of measured capacities during slush transfer.

The measurements and the acquisition of the capacity values were made with a LCR meter Keysight, model E4980AL, having the applied voltage of 1 V at 1 Mhz frequency, as recommended by Ohira and Nakamichi [23]. The evolution of the values of the two capacities, measured during the first slush nitrogen flow experiments, for one second, is shown in Figure 6.

The acquisition of the two signals, *C1* and *C2*, was made at 0.012 s intervals as series of values. To determine the delay time *τ*, we calculated the correlation index, *ρ_τ_*, for the two series, with the relation [24]:

(6) $$\rho_{\tau }=\frac{\sum_{i=0}^{N-1}\left[\left(x\left(i\right)-Mx\right)\cdot \left(y\left(i-\tau \right)-My\right)\right]}{\sqrt{\sum_{i=0}^{N-1}{\left(x\left(i\right)-Mx\right)}^{2}}\cdot \sqrt{\sum_{i=0}^{N-1}{\left(y\left(i-\tau \right)-My\right)}^{2}}},$$

where, *x*(*i*)—*C1* measured values series; *y*(*i*)—*C2* measured values series; *Mx*—arithmetic mean values of series *x*(*i*); *My*—arithmetic mean values of series *y*(*i*).

The values of *ρ_τ_* are in [−1, 1] interval, where values closer to 1 represents a large correlation of the two series and closer to 0 indicates that there is no correlation. The negative values closer to −1 indicates a large correlation, but with the inverse of one of the series.

**Figure 7:**
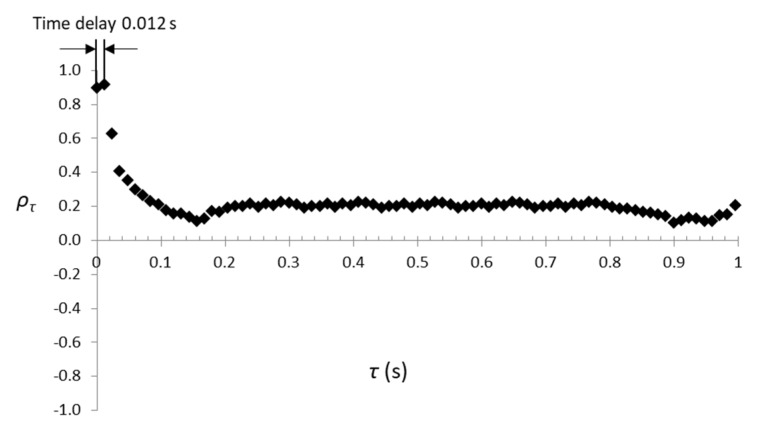
Calculated values of the correlation index for the *C1*, *C2* capacitors values during 1s slush nitrogen flow.

The calculated values *ρ_τ_* during 1 s slush nitrogen flow are presented in Figure 7. The maximum value of the correlation index (*ρ_τ_* = 0.91) was obtained for a delay *τ* = 0.012 s. This means that the second set of parameters is delayed compared to the first set with 0.012 s. Further, knowing that *v* = *l*/*τ* and using Equation (2) we calculated the mass flow as being 140.71 g/s (for a slush density *ρ_sl_* = 874 kg/m^3^ and solid volume fraction of *a_s_* = 30.56%).

**Figure 8:**
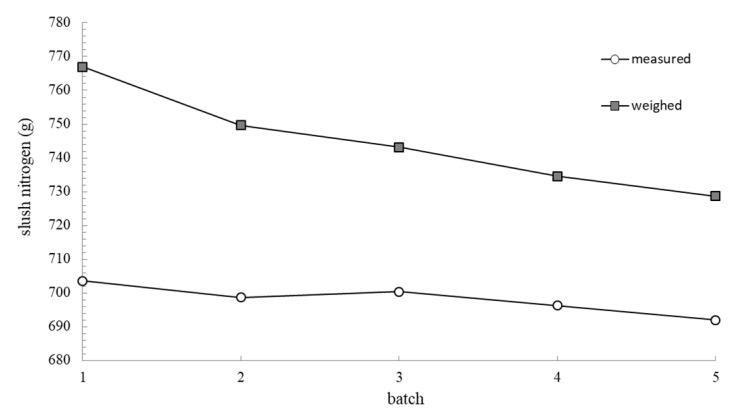
Measured and weighed values of the transferred slush.

Several tests for the production and transfer of the slush nitrogen through the experimental flowmeter were performed. The amount of slush nitrogen that passed through the flowmeter (measured using circular capacitors signals as exemplified above) was compared with the quantity of slush nitrogen transferred during one batch by weighing the producing Dewar vessel (M [g]). Figure 8 highlights the corresponding values of the estimated slush nitrogen quantity measured with flowmeter and the quantity measured by weighting, during five consecutive batches. Each transfer batch consists in delivery of the slush nitrogen through flowmeter for a limited time of 5 s. The experimental results are shown in Table 3.

Comparing the results presented in Table 3, it can be observed that the values obtained by weighing the Dewar vessel approximate the flow meter measured values, with an error of minimum 4.76%, except for the first batches where the error is higher. This phenomenon is due to a high rate (rates) of vaporization of slush nitrogen inside through the transfer line where, initially, the temperature is relatively higher in comparison with fluid that has been transferred. After the second batch, when the transfer line cooled to near the temperature of the slush nitrogen, the vaporization rate decreases significantly.

Apart from the errors due to the installation of the equipment, the accuracy depends also on the LCR capacity measurement accuracy (0.3%), weighing device (0.1%) and on the parasitic capacitance of the signal cables. To limit the parasitic capacitance of the cables influence on the capacitance measurement, the metallic shielding of the cables was connected to the LCR ground connection (with a length as short as possible).

## 4. Numerical Simulation

**Figure 9:**
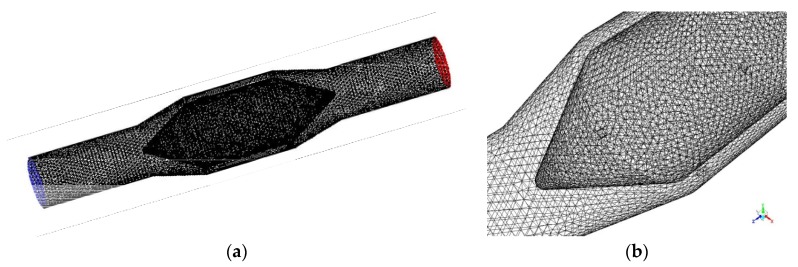
(**a**) Flowmeter mesh distribution; (**b**) Mesh distribution in the cone-shape flow restriction area.

In this research, a Eulerian–Eulerian model incorporating the kinetic theory of granular flow was used to simulate the nitrogen slush flow (two phase liquid–solid mixture) [25,26,27,28,29]. The governing equations used are presented in the Supplementary file. The Eulerian–Eulerian model handles the liquid and the solid phases as interpenetrating continua [26], in which the conservation equations (for momentum, mass, energy and turbulence) have similar structure for each phase and solved using the CFD approach. In this model, for the solid phase closure the kinetic theory of granular phase (inelastic collisions were considered) was used, in which the mean square of the solid particles velocity is defined as granular temperature (Θ) [27]. The turbulence model used was the dispersed k-ε model, which is the standard k-ε model with extra terms to include the turbulent interphase momentum transfer. The 3D model of the proposed flowmeter and mesh distribution is presented in Figure 9. The mesh was created using hexahedral cells criteria. The mesh density was examined by conducting a grid dependence test. Three different grids have been evaluated, from coarser to finer, using the refinement technique of the cells size near the walls and in the capacitor’s region (the flow restriction area), with an expand ratio of 1.01, 1.05 and 1.1, respectively. The value of y+ was compared and found that the results were between 15 and 120 for all three meshes, values acceptable for the k-e turbulence and flow conditions, with difference less than 0.6%. Therefore, to ensure the accuracy and to reduce the computational time, the medium grid, having 384,690 hexahedral cells (volumes), 1,025,742 tetrahedron-shaped surfaces and 401,872 nodes, was selected.

The model equations were numerically solved using the Fluent 13.0 software, developed by Ansys Inc., Canonsburg, PA, USA [30], by applying the implicit coupling pressure–velocity solver which is based on the “Phase Coupled SIMPLE” algorithm (it uses a relation between velocity and pressure corrections to impose the conservation condition of the mass and to obtain the pressure field), with second order discretization scheme for impulse, volume fraction and turbulence equations. The following boundary and initial conditions have been set: at the inlet (on the left side of the flowmeter), the liquid phase and the solid phase have the same velocity; at the walls no slip condition for liquid phase and Johnson–Jackson model for solid phase was used; at the outlet the final pressure condition was used *p_f_*; liquid nitrogen dynamic viscosity at NBP is 1.42 × 10^−4^ kg/m s; restitution coefficient *e_s_* = 0.9; granular temperature Θ = 0.0001 m^2^/s^2^; maximum solid volume fraction *a_s,max_* = 0.63; lift coefficient *C_lift_* = 0.25; specularity coefficient *ψ* = 0.02; turbulence model constants: *C*_1*ε*_ = 1.44, *C*_2*ε*_ = 1.92, σ*_k_* = 1, σ*_ε_* = 1.3, *C_µ_* = 0.09 [31]. 

Several simulations cases were carried out for slush nitrogen (liquid and solid mixture), with initial velocity *v*_0_ = 0.07 m/s and different values for final pressure, density of the mixture (corresponding to as volume fraction of the solids in the mixture) and dimensions of the solid particles. The detailed information of the simulated flow conditions are presented in Table 4. For each simulated case the solid particles are assumed to be spherical, inelastic and having the same size. The time step for all simulations was 0.005 s and the calculations were performed until a steady state of flow downstream of the flowmeter was obtained and the residuals amplitude decreases by at least five orders of magnitude. The average number of iterations for a case is 6000, corresponding to a 30 s mixture flow through the flowmeter.

### Simulation Results

The results obtained from the numerical simulations are illustrated below, comparatively presenting the profile of the mixing speeds or pressures under different flow conditions, as well as the variations of the maximum velocities of the liquid and solid phase, as a dependence on the size of the solid granules.

**Figure 10:**
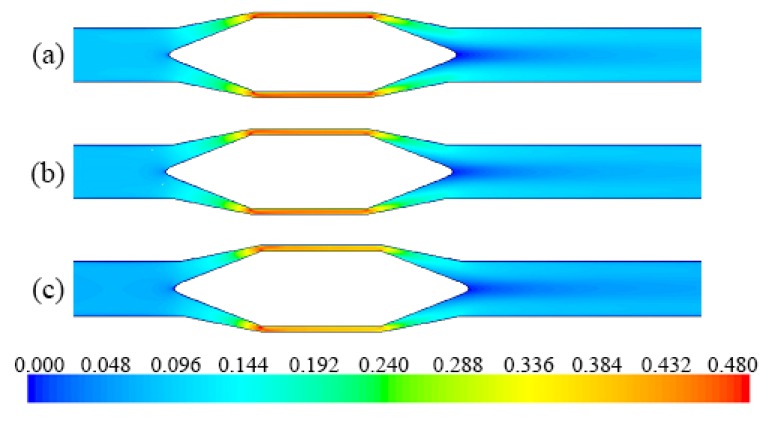
Liquid velocity profile (m/s): (**a**) case #1.1, *d* = 1 mm; (**b**) case #2.3, *d* = 1 mm; (**c**) case #3.4, *d* = 0.91 mm.

**Figure 11:**
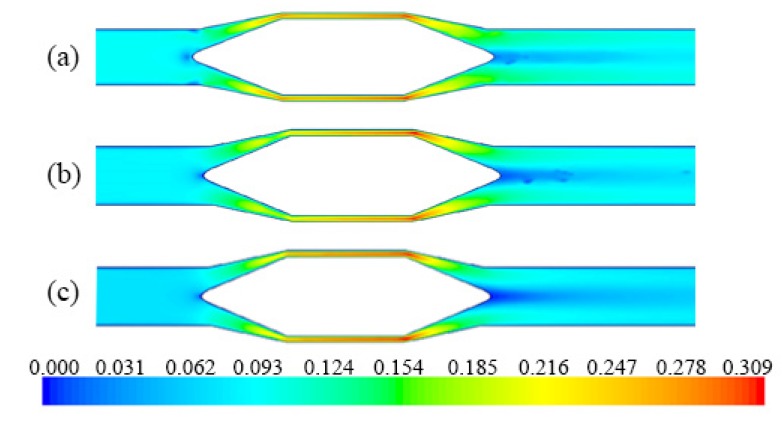
Solids velocity profile (m/s): (**a**) case #1.4, *d* = 1.34 mm; (**b**) case #3.4, *d* = 0.91 mm; (**c**) case #5.5, *d* = 0.54 mm.

From Figure 10 and Figure 11 it can be seen that the velocity of the liquid and solid phase through the flow section of the flowmeter increases with increasing of the mixture density or pressure. On the other hand, it is observed that the velocity profile at the output of the flowmeter returns, approximately, to the initial shape after a short section of the pipe compared with its diameter. This is a significant advantage of this configuration, the flowmeter can be mounted between straight sections of pipe, of lengths shorter than those needed for other flow measurement devices.

**Figure 12:**
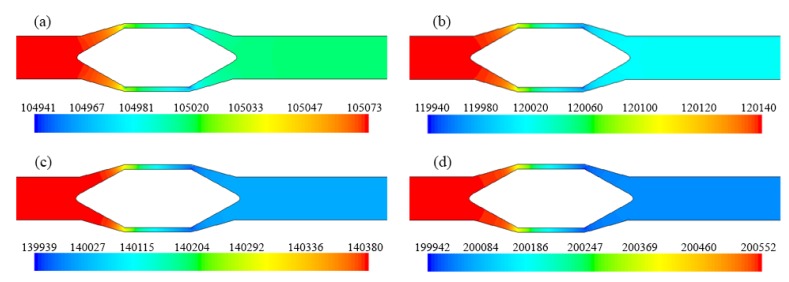
Absolute pressure (Pa) profile of the mixture: (**a**) case #1.1; (**b**) case #2.3; (**c**) case #4.4; (**d**) case #5.5.

From the absolute pressure profiles (Figure 12) a pressure drop is observed when passing the mixture through the flow meter, of about 0.27% for fluxes pressures greater than 140 kPa, which is not a big value for this type of flowmeters (with flow restriction).

**Figure 13:**
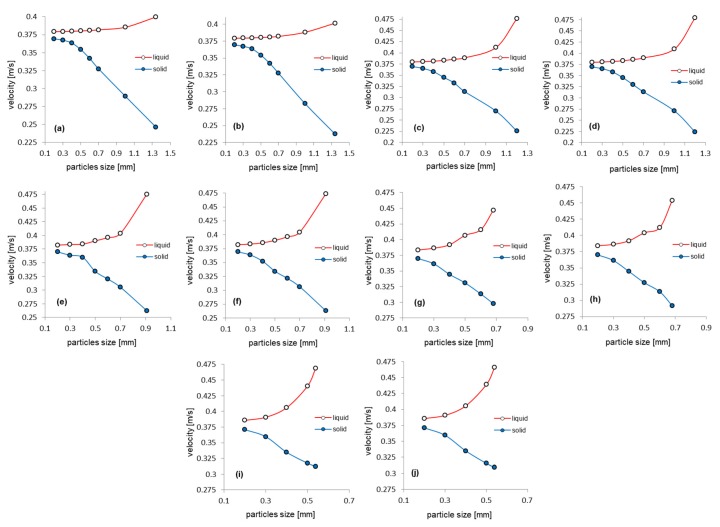
Maximum velocities of the liquid and solids depending on the solid particles size: (**a**) case #1.1; (**b**) case #1.5; (**c**) case #2.1; (**d**) case #2.5; (**e**) case #3.1; (**f**) case #3.5; (**g**) case #4.1; (**h**) case #4.5; (**i**) case #5.1; (**j**) case #5.5.

Figure 13 shows the variations of the maximum velocity of the liquid phase, respective of the solid phase, in the area of interest (flow restriction area), depending on the solid particles size. The maximum velocities characteristics of the liquid and solid phase have different shapes, the liquid phase velocity increases with increasing particle size, while the solid phase velocity decreases. In the cases with mixture densities of 830, 840 and 851 kg/m^3^ very small variations of the maximum velocities are observed, depending on the size of the granules, for pressures up to 200 kPa (a). In contrast, at higher densities (862 and 873 kg/m^3^), besides the particles size influence on the maximum velocities, the influence of pressure is also observed.

During the numerical simulations, a maximum limit of the size of the solid particles was observed, at different densities (Table 5) at which there was no flow through the flow meter (we can declare that the solution did not convert to a stable value), thus showing the limitations of the constructed model.

**Figure 14:**
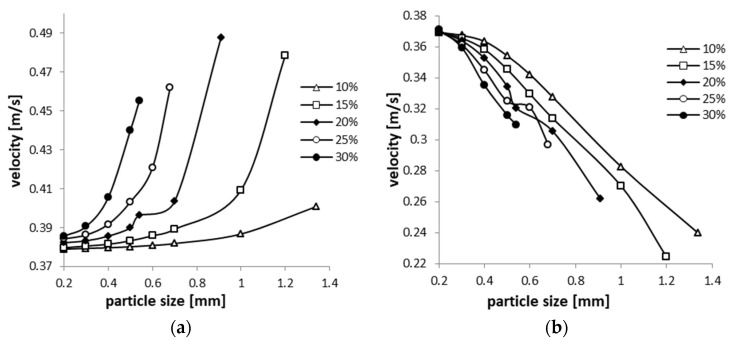
Liquid (**a**) and solid (**b**) phase velocity, depending on the solid particles size for different solid volume fractions at 140 kPa.

Figure 14 shows the variations of the liquid and solid phase maximum velocities, depending on the dimensions of the solid particles size, for different densities of the two-phase mixture. The data were represented up to the maximum grain size, according to Table 5. It can be observed that the velocity of the liquid phase increases, and that of the solid phase decreases, as the size of the granules increases. This phenomenon can be accounted for by the impulse exchange that occurs, in the flow process, between the solid and the liquid phase, practically set in motion by the solid phase. Additionally, in the same context, there is an increase of the differences in the flow velocity between the liquid phase and the solid phase, inside the flowmeter armatures, as the inlet pressure in the system is increasing.

**Figure 15:**
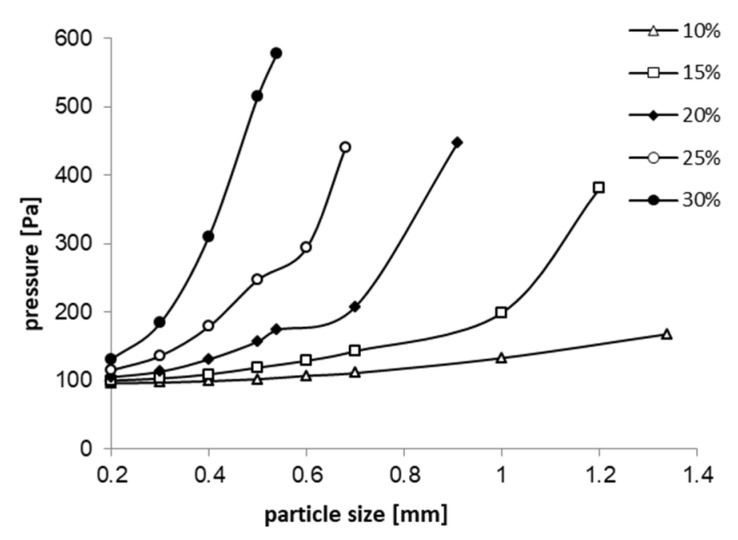
Pressure drop depending on the solid particles size for different solid volume fractions at 140 kPa.

Figure 15 graphically represents the pressure drops on the area of interest (the flow restriction area) according to the size of the granules, for different densities of the two-phase mixture (corresponding to a percentage mixture of 10%, 15%, 20%, 25% and 30% solid particles in the biphasic mixture. It can be observed that both the density of the mixture and the size of the solid particles directly influence the pressure drop. A percentage of solid phase of 30% and particle’s diameter of 0.54 mm is introducing a pressure drop of around 600 Pa along our flow meter, a value that is acceptable for a flow restriction device. For a bigger solid fraction, in order to have flow through the flowmeter, the size of the solid particles must be reduced further.

**Figure 16:**
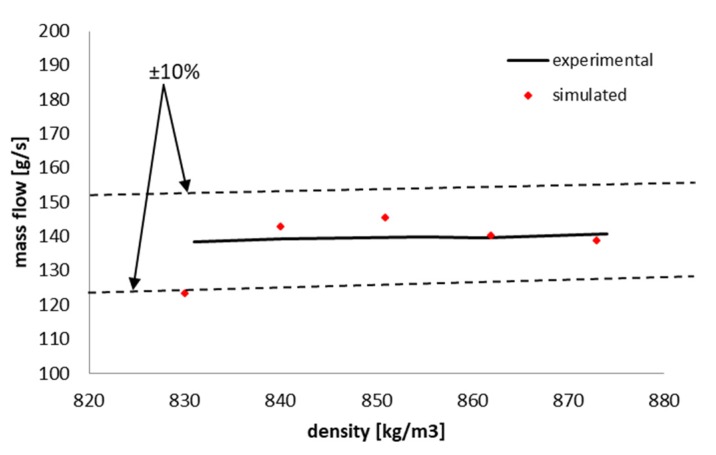
Experimental and numerical mass flow simulated for the densities and maximum solid particles stated in Table 5.

Figure 16 presents the experimental and numerical representation of the mass flow values of slush nitrogen flow passed through the new capacitive sensor of circular shape. 

The data were selected as follows:

- For the experimental data the values are represented for slush nitrogen densities as mentioned in Table 3,
- For the simulated mass flow data, the values presented are for the maximum particle sizes for each investigate density, as presented in Table 5.

It can be seen that simulated results agree well with the experimental results with a maximum deviation of ±10%.

## 5. Conclusions

Measuring the flow of solid–liquid mixtures of a cryogen (e.g., hydrogen, nitrogen) is quite difficult to perform. These special types of mixtures have special characteristics which imply the use of special measurement techniques. In the current market there is no such commercially available device; only devices created and used for different laboratories have been reported. 

In this paper we have presented a new design of capacitance type flow meter, which was experimentally tested for slush nitrogen flow, configuration which has also been numerically investigated using a CFD model. The capacitive measurement of slush-flow is advantageous due to the fact that it is not influenced by the pressure drop during the flow. The CFD model created for the numerical simulations was used for a better understanding of the phenomena that occur during the fluid flow through the inside of the capacitor.

The new design of circular shape capacitors took into account elements that force the flow and distribute the fluid evenly between the capacitor armatures, without requiring elements to calm the turbulence of the flow. Several tests for the production and transfer of the slush nitrogen through the experimental device were performed. The amount of slush nitrogen measured with the flowmeter, during a transfer batch (5s), was compared with the quantity difference measured by weighing the slush producing Dewar vessel. The calculated values with the flowmeter were higher than the weighed values with aproximately 5%–8%, due to the high rate (rates) of vaporization of slush nitrogen inside the transfer line between the pump and the flowmeter. To limit this error, the installation of the flowmeter has to be in the vicinity of the Dewar vessel in order to shorten this distance as the thermal insulation of the system has great importance for preserving the slush quality. Apart from the errors due to the installation of the equipment, the accuracy depends also on the capacity measuring setup. To limit the parasitic capacitance of the armatures signal cables, double shielded coaxial cables having low parasitic capacity (approximately 57 pF/m) and high accuracy LCR meter was used. The cables length was as short as possible and the metallic shielding of the cables were connected to the LCR ground connection. 

Several simulation conditions were performed for different flow conditions of the slush nitrogen, varying the mixture density, pressure and solid particles diameter. The simulated results agree well with the experimental results with a maximum deviation of ±10%. By comparing the results of the simulations performed, it was possible to conclude that the velocity profile of each phase is directly influenced by the mixture density and pressure. Additionally, a model limitation was noticed depending on the solid particle’s diameter, at different mixture densities. Solid phase above 30% should have particle diameters smaller than 0.54 mm in order to have flow through the proposed design flowmeter. The advantage of this configuration is the low pressure drop and that the velocity profile downstream of the flowmeter returns, approximately, to the initial shape after a short section of the pipe compared with its diameter. This will allow the flowmeter to be mounted between straight sections of pipe, of lengths shorter than those needed for other flow measurement devices. In this way the slush mixture can wash entirely and completely the desired surfaces that have to be cooled, eliminating the possibility of a clogged-up situation.

## Figures and Tables

**Table 1:** Thermophysical properties of para-hydrogen [17] and nitrogen [18] at normal boiling point (NBP) and triple point (TP).

		Temperature (K)	Pressure (kPa)	Density (kg/m^3^)	Dielectric Constant
para-hydrogen	NBP	20.271	101.325	70.828	1.230
	TP	13.8033	7.0410	76.977	1.252
nitrogen	NBP	77.35	101.325	808	1.457
	TP	63.14	12.53	1024	1.472

**Table 2:** Calibration of the capacitors *C1* and *C2*.

	*C1*	*C2*
air @294 K	14.01 pF	13.52 pF
nitrogen @NBP	19.24 pF	18.77 pF
nitrogen @TP	19.75 pF	19.28 pF

**Table 3:** Mass flow measured and weighed at different densities of the slush mixture.

Batch No.	1	2	3	4	5
*ρ*_*sl*_ [kg/m^3^]	874	861	855	840	831
*a*_*s*_ [%]	30.56	24.54	21.76	14.81	10.65
*Q_m_* [g/s]	140.71	139.73	140.08	139.24	138.39
M_measured_ [g]	703.59	698.67	700.41	696.24	691.99
M_weighed_ [g]	766.92	749.68	743.14	734.53	726.59
error	8.25%	6.8%	5.74%	5.21%	4.76%

**Table 4:** Simulated cases information. Each of the below cases were investigated for *v*_0_ = 0.07 m/s and different particle’s diameters between 0.2–1.4 mm

***ρ_sl_* [kg/m^3^] / *a_s._***	$p_{f}$ [kPa]					
		**105**	**110**	**120**	**140**	**200**
	**830/10%**	#1.1	#1.2	#1.3	#1.4	#1.5
	**840/15%**	#2.1	#2.2	#2.3	#2.4	#2.5
	**851/20%**	#3.1	#3.2	#3.3	#3.4	#3.5
	**862/25%**	#4.1	#4.2	#4.3	#4.4	#4.5
	**873/30%**	#5.1	#5.2	#5.3	#5.4	#5.5

**Table 5:** Maximum solid particles size for the flow condition.

density [kg/m^3^]	830	840	851	862	873
*d* [mm]	1.35	1.2	0.91	0.68	0.54

## Supplementary Materials

The Supplementary file are available online at https://www.mdpi.com/1424-8220/20/7/2117/s1.
