# Supplementary material for: Experimental Investigation and CFD Modeling of Slush Cryogen Flow Measurement Using Circular Shape Capacitors
Source: Sensors (Basel). 2020 Apr 9;20(7):2117. doi: 10.3390/s20072117 (PMC7181137; doi:10.3390/s20072117)
Supplement: Supplementary file 1 [file sensors-20-02117-s001.pdf]

sensors

MDPI

Governing Equations

Continuity equation for liquid and solid phase are:

$$\frac{\partial}{\partial t}(a_l \rho_l) + \nabla \cdot (a_l \rho_l \vec{v}_l) = 0 \quad (1)$$

$$\frac{\partial}{\partial t}(a_s \rho_s) + \nabla \cdot (a_s \rho_s \vec{v}_s) = 0 \quad (2)$$

$$a_l + a_s = 1 \quad (3)$$

Phase mass conservation equations:

$$\frac{\partial}{\partial t}(a_l \rho_l) + \nabla \cdot (a_l \rho_l \vec{v}_l) = \dot{m}_{sl} - \dot{m}_{ls} + S_l \quad (4)$$

$$\frac{\partial}{\partial t}(a_s \rho_s) + \nabla \cdot (a_s \rho_s \vec{v}_s) = \dot{m}_{ls} - \dot{m}_{sl} + S_s \quad (5)$$

The momentum equations are:

$$\frac{\partial}{\partial t}(a_l \rho_l \vec{v}_l) + \nabla \cdot (a_l \rho_l \vec{v}_l \vec{v}_l) = -a_l \nabla p + \nabla \overline{\overline{\tau}}_l + a_l \rho_l \vec{g} + \vec{F}_{sl} + \dot{m}_{sl} \vec{v}_{sl} - \dot{m}_{ls} \vec{v}_{ls} + \vec{F}_{lift,l} + \vec{F}_{vm,l} \quad (6)$$

$$\frac{\partial}{\partial t}(a_s \rho_s \vec{v}_s) + \nabla \cdot (a_s \rho_s \vec{v}_s \vec{v}_s) = -a_s \nabla p - a_s \nabla p_s + \nabla \overline{\overline{\tau}}_s + a_s \rho_s \vec{g} + \vec{F}_{ls} + \dot{m}_{ls} \vec{v}_{ls} - \dot{m}_{sl} \vec{v}_{sl} + \vec{F}_{lift,s} + \vec{F}_{vm,s} \quad (7)$$

$\vec{v}_{sl}, \vec{v}_{ls}$ are the interphase velocity, defined as:

if the solid phase mass is transferred to liquid phase ($\dot{m}_{sl}$ >0, $\dot{m}_{ls}$ <0) then $\vec{v}_{sl} = \vec{v}_{ls} = \vec{v}_s$;

if the liquid phase mass is transferred to solid phase ($\dot{m}_{sl}$ <0, $\dot{m}_{ls}$ >0) then $\vec{v}_{sl} = \vec{v}_{ls} = \vec{v}_l$;

The lift force, $\vec{F}_{lift,l} = -\vec{F}_{lift,s}$, is given by:

$$\vec{F}_{lift,l} = -C_{lift} \rho_l a_l (\vec{v}_l - \vec{v}_s) \cdot \nabla \vec{v}_l \quad (8)$$

Virtual mass force, $\vec{F}_{vm}$, appears when the secondary phase accelerates relatively to primary phase, and is given by [1]:

$$\vec{F}_{vm,l} = -\vec{F}_{vm,s} = 0.5 \rho_l a_s \left(\frac{d\vec{v}_l}{dt} - \frac{d\vec{v}_s}{dt} \right) \quad (9)$$

The interphase force:

$$\vec{F}_{sl} = -\vec{F}_{ls} = K_{sl} (\vec{v}_s - \vec{v}_l) \quad (10)$$

where K_{sl} is the liquid-solid exchange coefficient, determined by the Syamlal O'Brien model [2]:

$$K_{sl}=\frac{3}{4}C_D\frac{a_s a_l \rho_l}{v_{r,s}^2 d}\left(\frac{\mathrm{Re}_s}{v_{r,s}}\right)\left|\vec{v}_s-\vec{v}_l\right| \quad (11)$$

$$C_D=\left(0.63+\frac{4.8}{\sqrt{\mathrm{Re}_s/v_{r,s}}}\right)^2 \text{ [3]} \quad (12)$$

$$\mathrm{Re}_s=\frac{\rho_l d\left|\vec{v}_s-\vec{v}_l\right|}{\mu_l} \text{ [4]} \quad (13)$$

$$v_{r,s}=0.5\left(A-0.06\,\mathrm{Re}_s+\sqrt{(0.06\,\mathrm{Re}_s)^2+0.12\,\mathrm{Re}_s(2B-A)+A^2}\right) \text{ [5]}$$

$$A=a_l^{4.14} \quad (14)$$

$$B=\begin{cases}0.8a_l^{1.28}, a_l<0.85\\ a_l^{2.65}, a_l>0.85\end{cases}$$

Solids pressure is given by [6]:

$$p_s=a_s\rho_s\Theta+2\rho_s(1+e_s)a_s^2 g_{0s}\Theta \quad (15)$$

where g_{0s} is the radial distribution function [7]:

$$g_{0s}=\left[1-\left(\frac{a_s}{a_{s,\max}}\right)^{\frac{1}{3}}\right]^{-1} \quad (16)$$

Stress tensors for liquid and solid phase are defined by [8]:

$$\overline{\overline{\tau_l}}=a_l\mu_l\left(\nabla\vec{v}_l+\nabla\vec{v}_l^T\right)+a_l\left(\lambda_l-\frac{2}{3}\mu_l\right)\nabla\vec{v}_l\overline{\overline{I}} \quad (17)$$

$$\overline{\overline{\tau_s}}=a_s\mu_s\left(\nabla\vec{v}_s+\nabla\vec{v}_s^T\right)+a_s\left(\lambda_s-\frac{2}{3}\mu_s\right)\nabla\vec{v}_s\overline{\overline{I}} \quad (18)$$

The solid shear viscosity is:

$$\mu_s=\mu_{s,col}+\mu_{s,kin}+\mu_{s,fr} \quad (19)$$

where: $\mu_{s,col}$ is solids collisional viscosity [6,9]

$$\mu_{s,col} = \frac{4}{5} a_s \rho_s d g_{0s} (1 + e_s) \sqrt{\frac{\Theta}{\pi}} a_s \quad (20)$$

$\mu_{s,kin}$ is kinetic viscosity [10]:

$$\mu_{s,kin} = \frac{a_s \rho_s d \sqrt{\Theta \pi}}{6(3 - e_s)} \left[1 + \frac{2}{5}(1 + e_s)(3e_s - 1) a_s g_{0s} \right] \quad (21)$$

$\mu_{s,fr}$ is friction viscosity [11]:

$$\mu_{s,fr} = \frac{p_s \sin \phi}{2\sqrt{I_{2D}}} \quad (22)$$

Solid bulk viscosity is given by [6]:

$$\lambda_s = \frac{4}{3} a_s \rho_s d g_{0s} (1 + e_s) \sqrt{\frac{\Theta}{\pi}} \quad (23)$$

The conservation equation of the solids kinetic energy is [12]:

$$\frac{3}{2}\left[\frac{\partial}{\partial t}(a_s \rho_s \Theta) + \nabla \cdot (a_s \rho_s \Theta \vec{v}_s) \right] = \left(-a_s \nabla p_s \bar{\bar{I}} + \bar{\bar{\tau}}_s \right) : \nabla \vec{v}_s + \nabla \cdot (\kappa_s \Theta) - \gamma_s - 3K_{sl}\Theta + D_{ls} \quad (24)$$

where: κ_s is solid particles conductivity:

$$\kappa_s = \frac{25 \rho_s d \sqrt{\pi \Theta}}{64(1 - e_s) g_{0s}} \left[1 + \frac{6}{5}(1 - e_s) g_{0s} a_l \right]^2 + 2a_s^2 \rho_s d g_{0s} (1 - e_s) \sqrt{\frac{\Theta}{\pi}} \quad (25)$$

γ_s is solid particles dissipation rate:

$$\gamma_s = 3a_s^2 \rho_s g_{0s} \Theta (1 - e_s^2) \left(\frac{4}{d} \sqrt{\frac{\Theta}{\pi}} - \nabla \vec{v}_s \right) \quad (26)$$

D_{ls} is energy exchange rate:

$$D_{ls} = \frac{d \rho_s}{4 g_{0s} \sqrt{\pi \Theta}} \left(\frac{18 \mu_l}{d^2 \rho_s} \right)^2 |\vec{v}_l - \vec{v}_s|^2 \quad (27)$$

The turbulence model used, k-ε dispersed used is given by [13]:

$$\frac{\partial}{\partial t}(a_l\rho_l k)+\nabla(a_l\rho_l\vec{v}_l k)=\nabla\left(a_l\frac{\mu_t}{\sigma_k}\nabla k\right)+a_l G_k-a_l\rho_l\varepsilon-K_{sl}\left(2k-\sqrt{2k\cdot 3\Theta}\right) \tag{28}$$

$$\frac{\partial}{\partial t}(a_l\rho_l\varepsilon)+\nabla(a_l\rho_l\vec{v}_l\varepsilon)=\nabla\left(a_l\frac{\mu_t}{\sigma_\varepsilon}\nabla\varepsilon\right)+a_l\frac{\varepsilon}{k}(C_{1\varepsilon}G_k-C_{2\varepsilon}\rho_l\varepsilon)-C_{2\varepsilon}\frac{\varepsilon}{k}K_{sl}\left(2k-\sqrt{2k\cdot 3\Theta}\right) \tag{29}$$

$$\text{where: } \mu_t=a_l\rho_l C_\mu\frac{k^2}{\varepsilon} \tag{30}$$

turbulent viscosity,

$$G_k=\mu_t\left[\nabla\vec{v}_l\cdot\left(\nabla\vec{v}_l+(\nabla\vec{v}_l)^T\right)\right] \tag{31}$$

turbulent kinetic energy production rate

Johnson Jackson wall boundary condition [14]:

$$q_w-\frac{\sqrt{3}}{6}\pi\rho_s v_{slip}^2 g_{0s}\psi\frac{a_s}{a_{s,\max}}\sqrt{\Theta}+\frac{\sqrt{3}}{4}\pi\rho_s g_{0s}\frac{a_s}{a_{s,\max}}(1-e_w^2)\Theta^{3/2}=0 \tag{32}$$

Nomenclature

a—volume fraction

v—velocity, m/s

t—time, s

$\dot{m}_{sl}$—mass transfer from solid phase to liquid phase, kg

$\dot{m}_{ls}$—mass transfer from liquid phase to solid phase, kg

S—source term

p—total pressure, Pa

p_s—solids pressure, Pa

g—gravity acceleration, m/s^2

F_{ls}, F_{sl}—interphase interaction force, N

F_{lift}—lift force, N

F_{vm}—virtual mass force, N

$\vec{v}_{sl}, \vec{v}_{ls}$—interphase velocity, m/s

C_{lift}—lift coefficient

K_{sl}—liquid-solid transfer coefficient, kg/m^{3}s

C_D—drag coefficient

d—particles diameter, m

$v_{r,s}$—solid phase terminal velocity, m/s

Re_s—relative Reynolds number

g_{0s} —radial distribution function

e_s—restitution coefficient for interparticle collisions
e_w—restitution coefficient for particle-wall collisions
I—deviator stress tensor
D_{ls}—energy exchange rate, kg/m s^3
k—turbulent kinetic energy, m^2/s^2
$C_{1\varepsilon}$, $C_{2\varepsilon}$—turbulence model constants
C_μ —turbulent viscosity constant
G_k—turbulent kinetic energy production rate
q_w—solid particles energy near wall
v_{slip}—slip velocity

Greek leters
ρ—density, kg/m^3
τ—stress tensor, kg/s^2
κ_s —solid particles conductivity, kg/m^{3}s
μ—dynamic viscosity, kg/ms
λ—bulk viscosity, kg m/s
Θ —granular temperature, m^2/s^2
ϕ —internal friction angle
γ_s —solid particles disipation rate, kg/m s^3
ε—turbulent disipation rate, m^2/s^3
σ_k, σ_ε —turbulent Prandl numbers
ψ—specularity coefficient

Subscripts
l—liquid
s—solid
t—turbulent
vm—virtual mas

References

1. Drew, D.A.; Lahey, R.T. Analytical Modeling of Multiphase Flow. In: *Particulate Two-Phase Flow*, Butterworth-Heinemann, Boston, MA, USA, **1993**; pp. 509–566.
2. Syamlal, M.; O'Brien, T.J. Computer Simulation of Bubbles in a Fluidized Bed. *AIChE Symp. Ser.* **1989**, *85*, 22–31.

3. Dalla Valle, J.M. *Micromeritics*; Pitman: London, UK, **1948**.
4. Richardson, J.F.; Zaki, W.N. Sedimentation and Fluidization: Part I. *Trans. Inst. Chem. Eng.* **1954**, *32*, 35–53.
5. Garside, J.; Al-Dibouni, M.R. Velocity-Voidage Relationships for Fluidization and Sedimentation. *I & EC Process Des. Dev.* **1977**, *16*, 206–214.
6. Lun, C.K.K.; Savage, S.B.; Jeffrey, D.J.; Chepurniy, N. Kinetic Theories for Granular Flow: Inelastic Particles in Couette Flow and Slightly Inelastic Particles in a General Flow Field. *J. Fluid Mech.* **1984**, *140*, 223–256.
7. Lebowitz, J.L. Exact Solution of Generalized Percus-Yevick Equation for a Mixture of Hard Spheres. *Phy. Rev.* **1964**, *133*, 895–899.
8. Fluent, A. Ansys Fluent Theory Guide, ANSYS Inc., Canonsburg, PA, USA, 2013.
9. Chapman, S.; Cowling, T.G. *The Mathematical Theory of Non-Uniform Gases*. 3rd edition. Cambridge University Press: Cambridge, UK, **1990**.
10. Syamlal, M.; Rogers, W.; O'Brien, T.J.; *MFIX Documentation: Volume1, Theory Guide*. Report no. DOE/METC-9411004, National Technical Information Service: Springfield, VA, USA, **1993**.
11. Schaeffer, D.G. Instability in the Evolution Equations Describing Incompressible Granular Flow. *J. Diff. Eq.*, **1987**, *66*, 19–50.
12. Song, Y.; Zhu, J.; Zhang, C.; Sun, Z.; Lu, X. Comparison of liquid-solid flow characteristics in upward and downward circulating fluidized beds by CFD approach. *Chem Eng Sci* **2019**, *196*, 501–513.
13. Dadashi, A.; Zhu, J.; Zhang, C. A computational fluid dynamics study on the flow field in a liquid–solid circulating fluidized bed riser. *Powder Technol.* **2014**, *260*, 52–58.
14. Johnson, P.C.; Jackson, R. Frictional-collisional constitutive relations for granular materials, with applications to plane shearing. *J. Fluid Mech.* **1987**, *176*, 67–93.

© 2020 by the authors. Licensee MDPI, Basel, Switzerland. This article is an open access article distributed under the terms and conditions of the Creative Commons Attribution (CC BY) license (http://creativecommons.org/licenses/by/4.0/).
